# Antimicrobial resistance mechanisms in non-tuberculous mycobacteria

**DOI:** 10.1007/s12223-025-01287-z

**Published:** 2025-06-28

**Authors:** Esra Gül Tursun, Taylan Bozok, Gönül Aslan

**Affiliations:** Department of Medical Microbiology, Faculty of Medicine, Mersin University, Mersin, Türkiye

**Keywords:** Non-tuberculous mycobacteria, Antimicrobial resistance, Cell wall, Resistance mechanism

## Abstract

Non-tuberculous mycobacteria (NTM) are pathogens that are widely distributed in the environment and cause increasing rates of human infections. High levels of antimicrobial resistance shown by these bacteria complicate infection management and limit treatment options. The complex structure of cell walls and features such as biofilm formation are responsible for intrinsic resistance in NTMs. Antimicrobial resistance can be explained by four basic mechanisms: (i) limitation of drug uptake, meaning antibiotic entry is limited due to the presence of a hydrophobic and low permeability cell wall and a small number of porin channels, (ii) enzymatic modification of antibiotics, (iii) target site modification, (iv) efflux pumps, which prevent drug accumulation by actively expelling antibiotics from the cell and reduce treatment efficacy. For effective management of NTM infections, detailed understanding of resistance mechanisms, development of species-specific treatment protocols, and discovery of new antimicrobial agents are of great importance. In this review, the mechanisms causing drug resistance in NTMs will be reviewed.

## Introduction

Infections caused by nontuberculous mycobacteria (NTM) are increasing globally, and the natural resistance of these bacteria to many antibiotics complicates the treatment. NTMs are diverse and widespread in the environment, but only a few species cause serious and often opportunistic infections in humans. Recent epidemiological studies have shown that the incidence of lung disease caused by NTMs, in addition to *Mycobacterium tuberculosis* (MTB), is increasing at an alarming rate. Treatment options for NTM infections are limited due to high rates of antimicrobial resistance, and long treatment periods, risk of toxicity, and the development of tolerance further complicate infection management. The most frequently reported species as pulmonary pathogens among NTMs are *Mycobacterium avium* complex (MAC) and *Mycobacterium abscessus* complex (MABC). Since these species are specifically known to have high rates of resistance to commonly used anti-tuberculosis drugs, the need for species-specific treatment regimens is emphasized. *M. abscessus*, which is intrinsically resistant to many antimicrobials and has the lowest treatment success and remission rates, is often referred to as the “untreatable nightmare” (Tarashi et al. 2022; Wu et al. 2018).

Both intrinsic (natural) and acquired antimicrobial resistance mechanisms are prevalent in NTM populations. Intrinsic and inducible antimicrobial resistance mechanisms further complicate the demanding and individualized treatment regimens required to suppress infection. In addition to these resistance mechanisms, mycobacteria develop acquired resistance mechanisms through genomic mutations after long-term antimicrobial therapy (Gopalaswamy et al. 2020; Wu et al. 2018).

To effectively manage NTM infections, continuous surveillance, species-specific treatment protocols, and development of new antimicrobial agents are of great importance. Understanding the mechanisms of resistance is essential for the development of new treatments and prevention of the increasing prevalence of drug resistance in mycobacterial infections. This review aims to review the molecular mechanisms leading to resistance development in NTMs.

## Antimicrobial resistance mechanisms

**Fig. 1:**
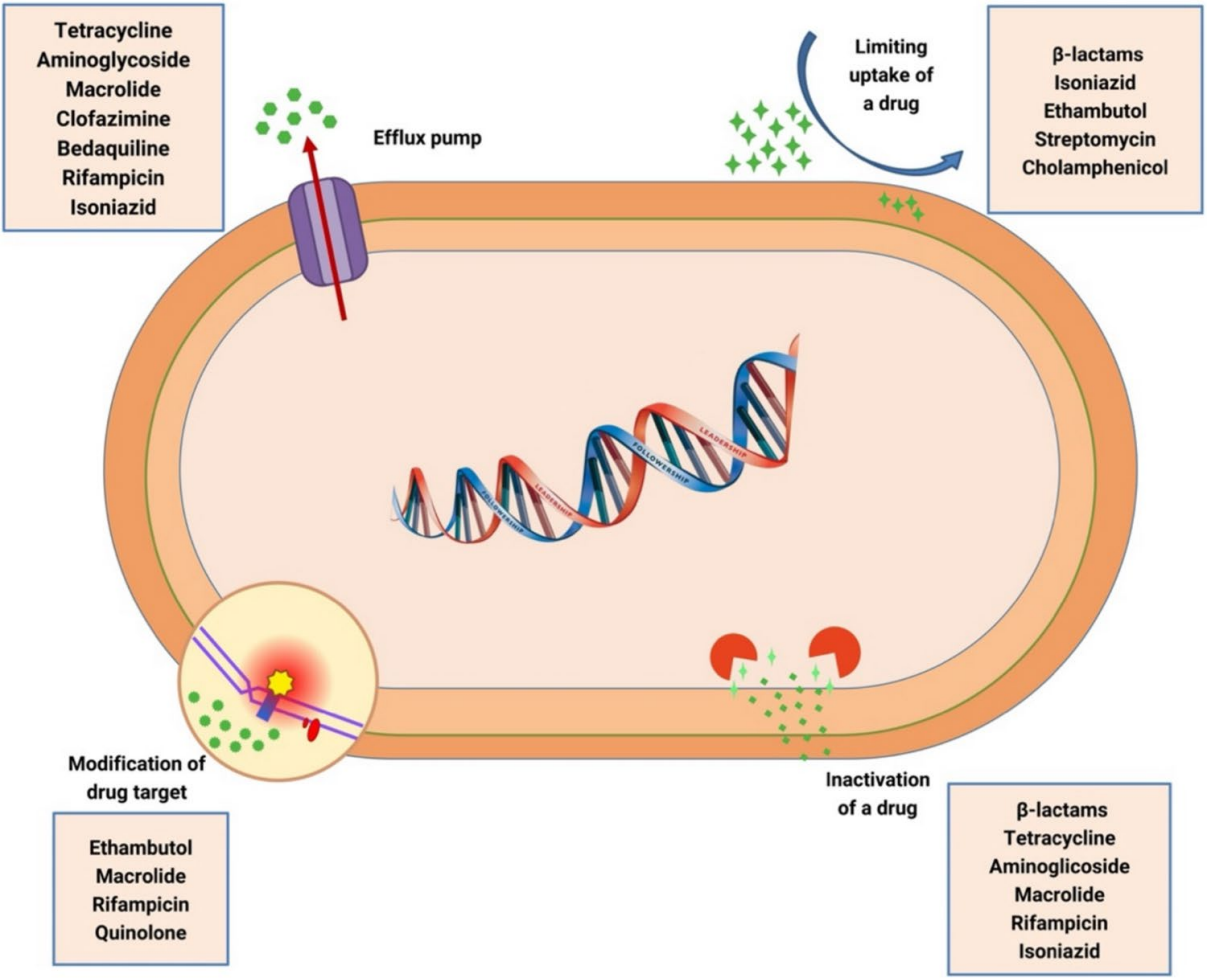
Antimicrobial resistance mechanisms

Antimicrobial resistance mechanisms can be classified under four main headings: limitation of drug uptake (decreased permeability), enzymatic modification of antibiotics, drug target site modification, and the presence of active efflux pumps (EPs) (Helmy et al. 2023) (Fig. 1).

## Limitation of drug uptake (decreased permeability)

One of the most important factors responsible for natural resistance in NTMs is their thick, hydrophobic, impermeable cell walls, which effectively reduce drug uptake. In addition to the cell wall, the low porin count of mycobacteria and their presence in biofilms and granulomas play a role in reducing drug concentration within the bacteria (Luthra et al. 2018; Saxena et al. 2021).

### Cell wall

**Fig. 2:**
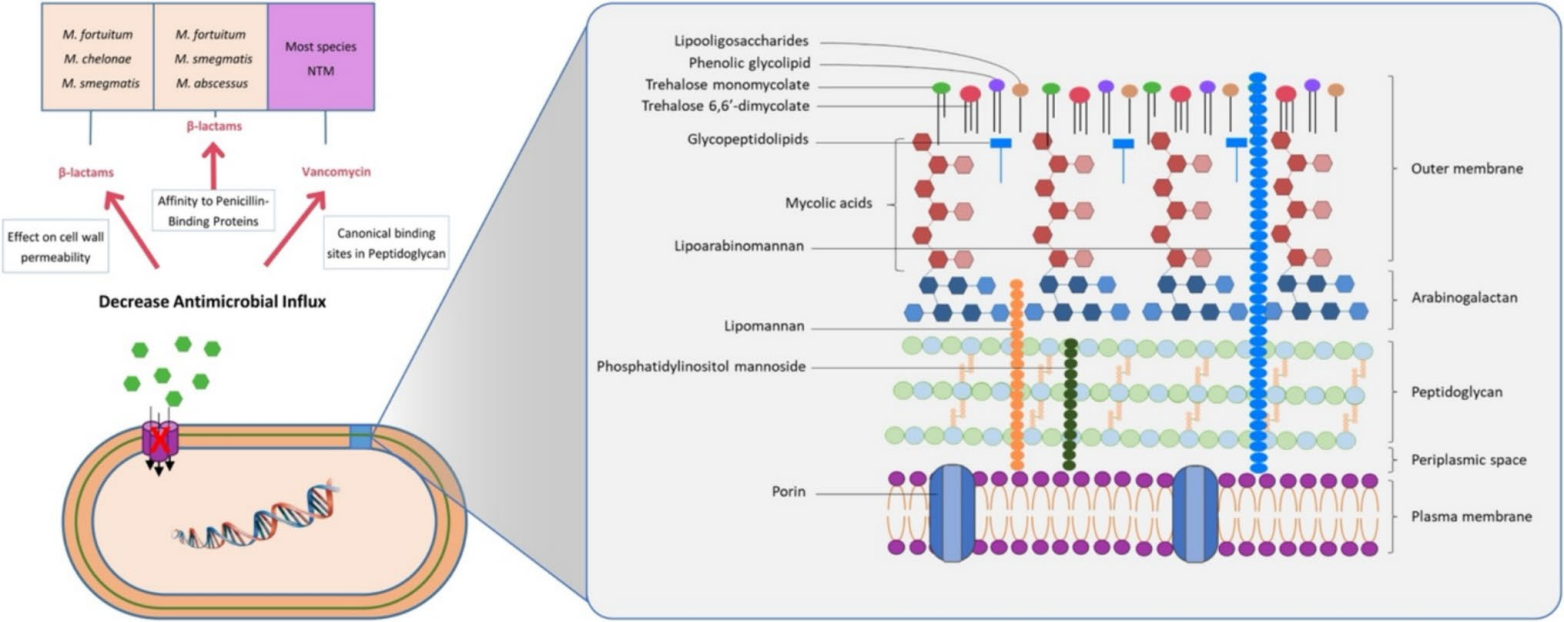
Cell wall structure of mycobacteria and its role in antimicrobial resistance

The extraordinary lipophilic cell wall structure of mycobacteria is one of the biggest obstacles to therapeutic intervention against these bacteria. The cell wall is a giant ternary complex consisting of mycomembrane (outer membrane), arabinogalactan (AG), and peptidoglycan (PG). Mycobacterial plasma membrane, which has similar properties to plasma membranes of other bacteria, is surrounded by a cross-linked PG layer. PG provides rigidity to the cell with its web-like structure and provides resistance to osmotic pressure by maintaining cell integrity. AG, the major cell wall polysaccharide, is covalently attached to PG. The outer ends of AG are esterified with mycolic acids (MA), which are high molecular weight fatty acids (Fig. 2). MA has strong hydrophobic properties and protects the bacillus against hydrophilic antibiotics and oxidative damage. PG, AG, and MA form the cell wall core structure, which is called the mycolyl-arabinogalactan-peptidoglycan (mAGP) complex. mAGP plays a critical role in the overall architecture of this unique cell wall and in regulating its permeability (Jacobo-Delgado et al. 2023; Singh et al. 2018; Tran et al. 2019). Mycobacterial outer membrane consists of two parts, inner and outer layer. The inner layer mainly contains MAs, while the outer layer consists of trehalose monomycolate, trehalose 6,6′-dimycolate (TDM), various glycolipids, phospholipids, and glycopeptidolipids (GPL). Phosphatidylinositol mannosides and their derivatives lipomannan (LM) and lipoarabinomannan (LAM) are membrane glycolipids bound with phosphatidylinositol found in the inner (plasma membrane) and outer membrane of the cell wall. LAM starts from the plasma membrane and extends along the cell wall (Fig. 2). LM is important for cell wall biogenesis and survival of bacilli within the host. LAM stabilizes the cell wall and regulates plasma membrane permeability. In addition, the presence of trehalose-containing lipooligosaccharides has been reported in some mycobacteria such as *M. canettii*, *M. Smegmatis*, and *M. gordonae* (Fig. 2) (Singh et al. 2018; Tran et al. 2019). Various enzymes involved in cell wall structure are important for the maintenance of the multidrug-resistant phenotype (van Ingen et al. 2012). Any defect in enzymes and proteins found in cell wall structure can lead to increased susceptibility to multiple drugs. MurA and MurB are enzymes involved in PG biosynthesis. The broad-spectrum antibiotic fosfomycin is a specific inhibitor of MurA and acts by forming a covalent bond with a cysteine ​​residue in the active site. MTB shows intrinsic resistance to fosfomycin by converting the cysteine ​​residue to aspartic acid. Antigen 85 (Ag85) complex proteins are involved in the synthesis of TDM, which is important for cell wall integrity in MTB. Inactivation of the Ag85 gene affects mycolate content and alters MTB cell wall permeability, resulting in increased susceptibility to both first-line tuberculosis (TB) drugs and other broad-spectrum antibiotics. Studies on Ag85 genes (*fbpA*,* fbpB*, and *fbpC*) have shown that mutant strains have 40% less mycolate in the cell wall. TDM production by Ag85 is required for intrinsic antibiotic resistance of mycobacteria, and the use of Ag85-specific inhibitors alone or in combination with other antibiotics may provide effective treatment for TB and other mycobacterial diseases (Nasiri et al. 2017; Singh et al. 2020).

Other important proteins known to play a role in mycobacterial cell wall integrity are protein kinase G (*pknG*), *asnB* (*M. smegmatis*), *mtrAB* (*M. smegmatis* and *M. avium*), *kasB* (*M. marinum*), and *pks12* (*M. avium*). For example, inactivation of *asnB*, which encodes asparagine synthase responsible for amino acid metabolism, disrupts cell wall structure and thus causes hypersensitivity to hydrophobic drugs in *M. smegmatis*. Similarly, *pknG* plays an important role in intrinsic resistance to multiple antibiotics in mycobacteria by controlling cell wall structure. Disruption of these genes generally reduces the hydrophobicity of the mycobacterial cell wall and leads to increased susceptibility to lipophilic antibiotics, including rifamycins, macrolides, ciprofloxacin, vancomycin, imipenem, and penicillins (Saxena et al. 2021; van Ingen et al. 2012). In addition to proteins involved in metabolism, the activity of proteins directly involved in cell wall structure, such as *mtrAB* and *fbpa*, greatly influences the sensitivity of mycobacteria to hydrophilic drugs. Deletion of the *fbpa* gene renders *M. smegmatis* sensitive not only to hydrophilic antibiotics but also to hydrophobic ones due to increased cell wall fluidity (Saxena et al. 2021).

NTMs exhibit two different colony morphologies, smooth colony morphotype (S = smooth) and rough colony morphotype (R = rough). These differences in morphotypes are related to the high or low GPL content in the cell wall, respectively. This distinctive feature, which is associated with the GPL status, affects biofilm formation and drug sensitivity (Nguyen et al. 2024). Smooth colony morphotypes are more sensitive to ciprofloxacin, clarithromycin (CLA), and penicillin. This increased sensitivity is regulated by the *mtrAB* two-component system (van Ingen et al. 2012).

Intrinsic resistance to β-lactam antibiotics may occur due to an impermeable cell wall, reduced affinity for penicillin-binding proteins (PBPs), and activity of β-lactamases (Fig. 2). Vancomycin acts by inhibiting the formation of mature peptidoglycans, but most NTM species are intrinsically resistant to this agent. The best-known mechanism of vancomycin resistance is the presence of VanA or VanB; however, this has not been described for NTM species. Vancomycin resistance in NTMs is due to the presence of specific conserved canonical binding sites for vancomycin in mycobacterial peptidoglycan (Fig. 2) (Tarashi et al. 2022).

### Porin deficiency

The permeability of the mycobacterial cell wall to hydrophilic substances is extremely low compared to other bacteria. The hydrophobic cell wall also makes it difficult for the bacilli to take in nutrients from the environment. For this reason, mycobacteria use channel proteins called porins for the uptake of nutrients and the passage of hydrophilic agents (Fig. 2). Unlike other bacteria, the number and permeability of porins in mycobacteria are quite low. While small hydrophilic antibiotics such as β-lactams can access mycobacteria via porins, the entry of other small hydrophilic agents is quite limited due to the high specificity of porins (Tran et al. 2019). In mycobacteria, only *mspA*, the major porin of *M. smegmatis*, has been studied extensively, and its activity determines susceptibility to norfloxacin, chloramphenicol, and β-lactam and hydrophilic antibiotics such as isoniazid (INH), ethambutol (EMB), and streptomycin (SM). Deletion of *mspA* significantly increases the resistance of *M. smegmatis* to these hydrophilic agents (Nasiri et al. 2017; van Ingen et al. 2012).

### Biofilm ve granulomas

NTMs form biofilms in environments such as water pipes, shower heads, and surfaces of healthcare equipment (Saxena et al. 2021). During biofilm development, bacteria undergo phenotypic changes to form a heterogeneous, dynamic, and differentiated community (Faria et al. 2015). Bacteria within biofilms exhibit enhanced resistance to antimicrobial agents and disinfectants, and this resistance can be 10 to 1000 times higher than planktonic forms. The waxy, lipid-rich extracellular polymerized substances (EPS) of the biofilm are thought to form a strong physical barrier that prevents drug penetration (Faria et al. 2015; Saxena et al. 2021). GPL, found in the cell wall structure of NTMs, affects biofilm formation, substance composition, and structure. During biofilm formation, genes related to GPL biosynthesis are upregulated in *M. avium*,* M. abscessus*, and *M. smegmatis*, indicating that GPL synthesis and biofilm formation are closely related (Faria et al. 2015).

S morphotype strains, such as *M. abscessus* and *M. bolletii*, which contain abundant GPL in their cell wall structure, are more prone to in vitro biofilm formation. Decreased susceptibility to several first-line antibiotics, such as cefoxitin, amikacin, and CLA, has been shown in biofilm models (Nguyen et al. 2024).

Granuloma is one of the host defense mechanisms and immunological distinguishing features that prevent the spread of bacilli. While granuloma supports host defense by limiting the spread of bacilli, it also protects the pathogen by limiting drug penetration into the bacteria within the granuloma structure. Anoxic conditions in the granuloma center stimulate physiological and morphological mechanisms that make mycobacteria more tolerant. Although granulomas are considered a host defense structure aimed at eliminating the pathogen, mycobacteria can survive in a latent state within the granuloma for years. It is known that MAC and *M. abscessus* in particular can persist in granulomas for several years or decades without showing symptoms. However, NTMs have not been shown to cause latent infection. Therefore, unlike TB, there is currently no evidence that NTMs are associated with reactivation disease (Broncano-Lavado et al. 2022; Griffith et al. 2007; Saxena et al. 2021).

## Enzymatic modification of antibiotics

Mycobacteria produce enzymes that degrade, modify, or inactivate different classes of antibiotics, including β-lactams, aminoglycosides, and macrolides. β-lactam antibiotics act by inhibiting bacterial transpeptidases responsible for the final step of PG cross-linking. β-lactam resistance in mycobacteria results from production of the Ambler class A β-lactamase, encoded by the *blaC* gene. Examples of these enzymes in NTMs include the major β-lactamase, *blaS*, and the cephalosporin-specific β-lactamase, *blaE*. Due to the presence of β-lactamases, NTMs exhibit high levels of innate resistance to most β-lactams, except cefoxitin and imipenem (Nasiri et al. 2017; Sachan et al. 2023; Wu et al. 2018).

Rifampicin (RIF), which is frequently used as a first-line agent in the treatment of MTB infections, has limited efficacy in NTMs. Rifamycin group antibiotics bind to the β-subunit of bacterial RNA polymerase and inhibit transcription. Resistance to rifamycins is often associated with mutations in the *rpoB* gene, which encodes the β-subunit of RNA polymerase. However, the main mechanism responsible for resistance to rifamycins in NTMs is enzymatic inactivation of the drug by ADP-ribosyltransferases (*Arr*) (Luthra et al. 2018; Saxena et al. 2021).

Another first-line TB drug, INH, is a prodrug, meaning it requires bacterial catalase peroxidase (*katG* enzyme) to become active. Decreased catalase/peroxidase activity due to katG mutations is the most common mechanism associated with INH resistance. EP are also responsible for INH resistance. Most NTM species are naturally resistant to INH due to lack of the *katG* enzyme (Nasiri et al. 2017; Tarashi et al. 2022).

The aminoglycoside class of antibiotics inhibits protein synthesis by binding with high affinity to the 16S rRNA A region of the bacterial 30S ribosomal subunit. Aminoglycosides commonly used in NTM treatment regimens include amikacin, SM, gentamicin, and tobramycin. Drug and target site modification is the most clinically important aminoglycoside resistance mechanism that has been extensively studied in mycobacteria (Luthra et al. 2018). Drug resistance to aminoglycosides is mainly associated with point mutations in the *rrs* gene. Long-term use of aminoglycosides leads to the emergence of spontaneous mutations in the *rrs* gene encoding the 16S rRNA protein (Johansen et al. 2020). *rrs* mutations have led to resistance to aminoglycosides such as amikacin in *M. abscessus* and *M. chloenae* (Victoria et al. 2021). Two major classes of aminoglycoside-modifying enzymes, N-acetyltransferases and phosphotransferases, have been demonstrated in various mycobacterial species (Nasiri et al. 2017). Phosphotransferases and N-acetyltransferases contribute to drug resistance by modifying the hydroxyl and amino groups in aminoglycosides, respectively (Johansen et al. 2020). Different N-acetyltransferases have been identified in the genomes of *M. kansasii*, the fast-growing *M. fortuitum*,* M. smegmatis*, and *M. abscessus* (Nasiri et al. 2017). Phosphotransferases have been identified in *M. fortuitum*,* M. abscessus*, and *M. avium* (Tarashi et al. 2022).

Tetracyclines bind to the 30S subunit of bacterial ribosomes and prevent the delivery of aminoacyl-tRNA to the ribosomal A site, thereby inhibiting bacterial protein synthesis. The mechanisms responsible for tetracycline resistance in mycobacteria are EPs, ribosomal protection proteins (*otrA*), and the enzymatic inactivation of tetracyclines. *TetV/Tap* EP is responsible for low-level intrinsic resistance to tetracyclines in *M. smegmatis* and MTB. *M. abscessus* is 500-fold more resistant to tetracycline and doxycycline than *M. smegmatis* and MTB. The mechanism responsible for this resistance is the inactivation of tetracyclines by the flavin-adenine-dinucleotide (FAD)-dependent monooxygenase, MabTetX (Luthra et al. 2018; Rudra et al. 2018). However, the tetracycline derivative tigecycline is resistant to enzymatic inactivation, and *M. abscessus* is sensitive to tigecycline in vitro (Victoria et al. 2021).

## Target site modification

Some NTM species have target gene polymorphisms that contribute to drug resistance. For example, amino acid changes in the *embB* gene, which encodes arabinosyltransferases in *M. abscessus*, render EMB inactive by preventing drug binding (Wu et al. 2018). Mutations in the *embCAB* operon encoding arabinosyltransferases (especially the *embB* gene) are the main cause of EMB resistance in MTB (Nasiri et al. 2017).

In some NTM species, drug exposure induces expression of specific genes, resulting in modification of the drug’s target binding site. A good example is the induction of the *erm41* gene, which encodes erythromycin ribosome methylase (*erm*), in *M. abscessus* by exposure to macrolides. Exposure to CLA or azithromycin significantly increases *erm41* gene expression within 24 h (Wu et al. 2018).

Macrolides bind to the 23S rRNA unit of the 50S subunit of the bacterial ribosome and stop protein synthesis. Methylation of 23S rRNA by *erm4*1 reduces the binding affinity of macrolides to the ribosome and causes macrolide resistance. Deletion of the erm41 gene in *M. abscessus* reduced the CLA minimum inhibitory concentration (MIC) value by 128-fold (Luthra et al. 2018) In addition, mutations in the *rrl* gene encoding 23S rRNA peptidyl transferase in mycobacteria are associated with acquired macrolide resistance (Victoria et al. 2021).

Another mechanism effective in RIF resistance in mycobacteria is RNA polymerase binding protein A (*RbpA*), which has been identified especially in MTB and *M. smegmatis*. This protein prevents RIF from binding to RNA polymerase by causing a structural change in the binding site. *RbpA* causes RIF resistance by binding to RNA polymerase (Nasiri et al. 2017; Saxena et al. 2021).

Another example of genetic polymorphism affecting drug resistance is fluoroquinolones. Quinolones are secondary therapeutic agents for multidrug-resistant TB (MDR-TB) and they target two essential bacterial topoisomerases, DNA gyrase (also known as topoisomerase II) and DNA topoisomerase IV. These enzymes regulate DNA supercoiling and are therefore essential for bacterial DNA replication and cell division. Fluoroquinolones potently inhibit the supercoiling activity of DNA gyrase and topoisomerase. The primary mechanism responsible for quinolone resistance is polymorphisms and point mutations in the *gyrA* and *gyrB* genes encoding DNA gyrase (Nguyen et al. 2024; Tarashi et al. 2022). Target site modification has also been associated with quinolone resistance. An example of target site modification is the *mfpA* gene, which confers resistance to quinolones in MTB. The DNA gyrase-binding protein *mfpA* encodes pentapeptide repeat proteins that bind to DNA gyrase by mimicking the DNA B form in size and shape and protects it from the lethal effects of quinolones (Nasiri et al. 2017).

Linezolid is the first member of the oxazolidinone group of antibiotics. It binds to the 50S subunit of ribosomes in bacteria and inhibits protein synthesis. Linezolid, the main oxazolidinone currently in clinical use, is used primarily in the treatment of drug-resistant TB. It is known to have some in vitro activity against MAC and *M. abscessus* and is recommended for the treatment of these species. However, its clinical use is limited due to toxicity concerns. Tedizolid was developed as an alternative agent with a lower side effect profile compared to linezolid. Mutations in the *rplC* gene, which encodes 50S ribosomal protein L3, and the *rrl* gene, which encodes 23S rRNA, have been detected in linezolid-resistant MTB clinical isolates (Nasiri et al. 2017). However, the resistance mechanism, efficacy, and tolerability of linezolid and tedizolid have not yet been sufficiently investigated for NTM isolates. A phase IV clinical trial is ongoing in Bangkok to investigate the efficacy and tolerability of linezolid in the treatment of NTM diseases (Nasiri et al. 2017; Wu et al. 2018).

## Efflux pumps (EP)

EPs have been identified as another mechanism contributing to antibiotic resistance in mycobacteria. The primary role of EP systems is to protect bacteria from harmful substances, maintain cellular homeostasis, and maintain physiological balance by excreting toxins or metabolites into the extracellular environment (Nguyen et al. 2024). EPs contribute to the intrinsic resistance of mycobacteria to many drugs by actively excreting many antibiotics from the cell and preventing intracellular accumulation of antibiotics (Nasiri et al. 2017). EPs are transmembrane proteins encoded by genes and their expression can be induced. It has been suggested that antimicrobial use in mycobacteria induces EP genes and leads to chromosomal mutations in drug target genes along with drug efflux. Thus, EPs cause both intrinsic and acquired resistance (Schmalstieg et al. 2012).

**Fig. 3:**
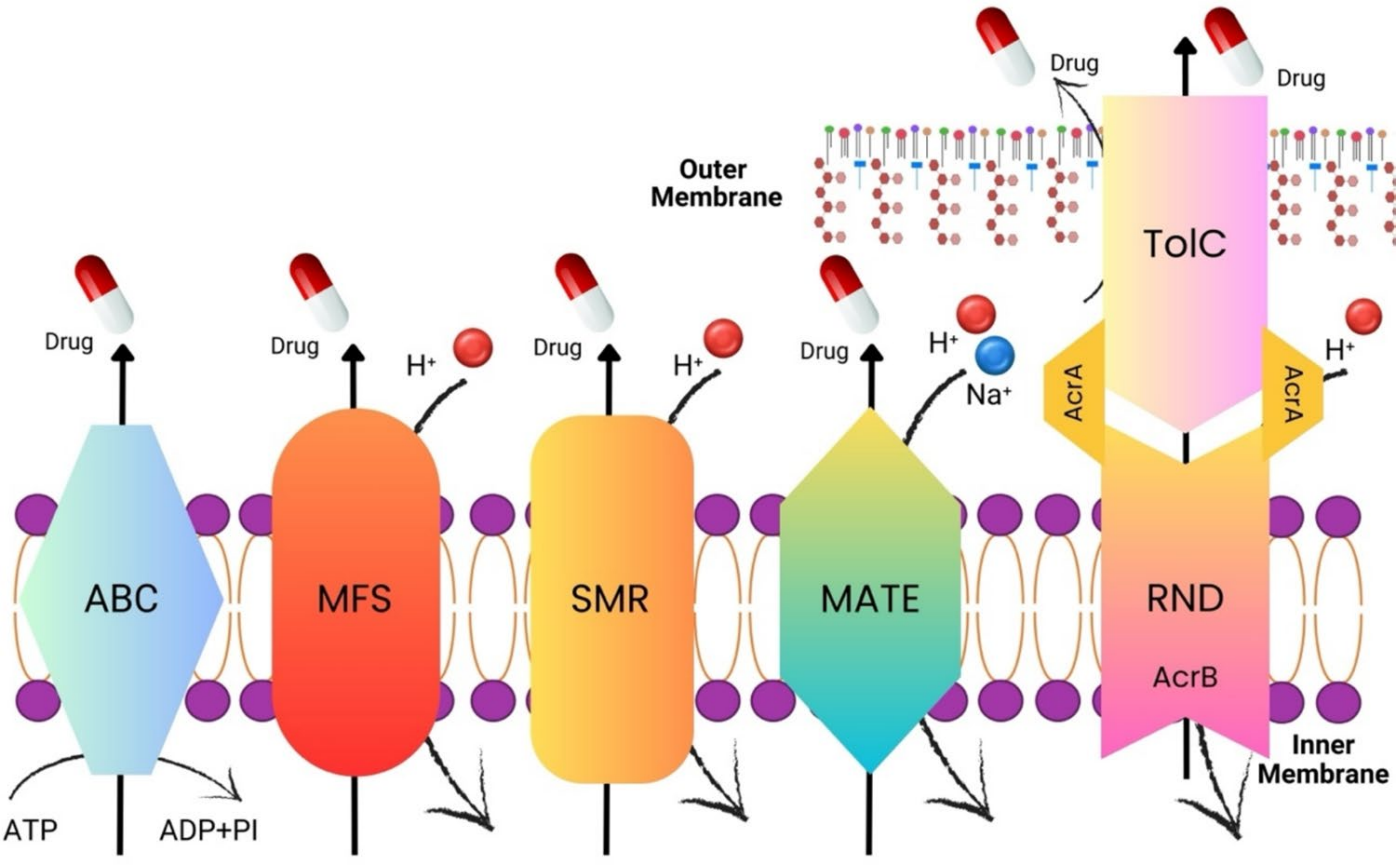
Schematic diagram showing the five EP superfamilies found in mycobacteria. EPs typically transport substrates across the cytoplasmic membrane to the extracellular environment. RND-type EPs are organized into trio systems and can transport substrates from the periplasm and cytoplasm across the outer membrane to the outside of the cell

EP systems are divided into five superfamilies according to their energy usage and structural features. These are “ATP-binding casette” ABC, “major facilitator superfamily” MFS, “Multidrug and Toxic Compound Extrusion” MATE, “small multidrug resistance” SMR, and “resistance nodulation division” RND. EPs included in the ABC superfamily are classified as primary transporters because they use ATP (adenosine triphosphate) as an energy source, while other EP superfamilies are classified as secondary transporters because they work with an electrochemical gradient and use protons (Fig. 3) (AlMatar et al. 2021).

The ABC superfamily is a group of transmembrane proteins consisting of a channel structure and at least two intracellular nucleotide-binding domains with cytosolic ATP-binding sites (Song and Wu 2016). ABC transporters play a role in antibiotic resistance, nutrient uptake, toxin removal, and mycobacterial virulence and pathogenesis. Human P-glycoprotein, encoded by *MDR1*, a protein belonging to the ABC family, is an EP found in many healthy tissues such as the gastrointestinal tract, liver, kidney, and blood–brain barrier, removing xenobiotics such as toxins and drugs from cells. Overexpression of P-glycoprotein in tumor cells has been associated with resistance to chemotherapeutics (De Rossi et al. 2006; Skinner et al. 2023).

The MFS superfamily is the largest family of secondary transporters and is found in the cytoplasmic membrane. They are responsible for the transport of various molecules such as simple sugars, amino acids, oligosaccharides, and other compounds, as well as providing antibiotic resistance (Alav et al. 2018).

The RND superfamily includes mycobacterial membrane proteins (*MmpL*) and mycobacterial small membrane proteins (*MmpS*). *MmpL* genes are associated with drug resistance in MTB. Studies have shown that *mmpL7* is an EP gene that causes INH resistance, and *mmpL7* can be antagonized by reserpine and carbonyl cyanide m-chlorophenyl hydrazone (CCCP), which are both EPs (Song and Wu 2016).

The genome of MTB contains genes encoding EPs belonging to all five superfamilies. Several ABC superfamily transporters such as *Rv0194*,* Rv1819c* (*BacA*), and *Rv2936/Rv2937/Rv2938* (*DrrABC*) have been shown to confer resistance to tetracycline, chloramphenicol, and various macrolides in MTB. The *Rv1456c*,* Rv1457c*, and *Rv1458c* pumps belonging to the ABC superfamily are associated with resistance to first-line drugs INH, RIF, EMB and SM in MTB. *Rv1258c*, also known as *Tap*, a member of the MFS family, can actively export the first-line TB drugs INH and RIF. Transcription of *jefA*, another member of the MFS family, leads to increased resistance to INH, EMB, and SM (Laws et al. 2022; Nasiri et al. 2017). Experimental overexpression of *rv0676c/rv0677c* (*MmpS5/MmpL5*), a transporter belonging to the RND superfamily, results in increased resistance to second-line antibiotics, including clofazimine and bedaquiline (BDQ) (Laws et al. 2022).

The presence of EPs has also been shown in NTMs. The best-defined EPs in NTMs are those that confer resistance to tetracycline and aminoglycosides in *M. fortuitum*, resistance to fluoroquinolones, RIF, and INH in *M. smegmatis*, resistance to macrolides in MAC, and resistance to clofazimine and BDQ in *M. abscessus* (Rindi 2020). *M. smegmatis* is 300 times more resistant to INH than MTB. This is due to efflux-related mechanisms. The much higher expression of *MmpL7* in *M. smegmatis* compared to MTB explains INH resistance (Louw et al. 2009). *Tap*, which confers resistance to tetracycline and aminoglycosides in *M. fortuitum*, and *LfrA*, a member of the MFS family that mediates fluoroquinolone, RIF, and INH resistance in *M. smegmatis*, are some of the first EPs identified in NTMs (Nasiri et al. 2017).

There are many EPs belonging to the *MmpL* family in *M. abscessus*. Increased expression of the *MmpS5/MmpL5* complex in *M. abscessus* is associated with clofazimine and BDQ resistance. In addition, a study has determined that three EP systems (ATP-binding cassette *MAB 2355c*, *MAB 1409c*, and *MAB 1846*) and their positive regulator gene *whiB7* contribute to CLA resistance in *M. abscessus* (Rindi 2020).

Similar to *M. abscessus*, EPs such as *MAV 1406* and *MAV 3306* have been implicated in macrolide resistance in MAC strains (Gutiérrez et al. 2019; Johansen et al. 2020).

The antimicrobial resistance mechanisms of NTM are summarized in Table 1.

**Table 1:** Antimycobacterial drugs and resistance mechanisms (Nasiri et al. 2017; Tarashi et al. 2022)

	Antibiotic	Mechanism of action	Resistance mechanism
Inhibition of cell wall synthesis	Isoniazid	Inhibition of mycolic acid synthesis	Mutation/polymorphism in *katG*
			Efflux pumps
	Ethambutol	Inhibition of arabinogalactan synthesis	Polymorphism/mutation in the target gene *embB*
	β-Lactams	Suppression of PBP enzymatic activity and peptidoglycan synthesis	Drug inactivation by β-lactamase-encoding genes (*Bla*)
	Vancomycin	Suppression of mature peptidoglycan assembly	Enhance cell wall permeability
	Delamanid	Suppression of keto- and methoxy-mycolic acid synthesis	Not determined for NTM species
Inhibition of DNA synthesis	Fluoroquinolones	Inhibition of DNA gyrase	Polymorphisms/mutations in the target genes *gyrA* and *gyrB*
			Target site modification by *mfpA*, a DNA gyrase-binding protein
			*LfrA* efflux pump
	Macrolides	Binding to 23S rRNA and inhibition of protein synthesis	Modification of the drug target by *erm41*
			Mutation in the 23S rRNA *rrl* gene
			Efflux pump
	Oxazolidinones (linezolid, tedizolid)	Binding to 23S rRNA and inhibition of protein synthesis	Mutation in the 50S ribosomal protein L3 (*rplC* gene) and the 23S rRNA *rrl* gene
Inhibition of protein synthesis	Aminoglycosides	Binding to 16S rRNA and inhibition of protein synthesis	Mutation in the 16S rRNA *rrs* gene
			Drug inactivation by phosphotransferase and acetyltransferase
	Tetracyclines	Binding to the 30S ribosomal subunit and inhibition of protein synthesis	Enzymatic inactivation by flavin-adenine dinucleotide (FAD)-inactivating monooxygenase (*TetX*)
			Activity of ribosome protection proteins (*otrA* and *tetM*)
			*TetV/Tap* efflux pump
	Rifampicin	Inhibition of transcription by binding to the β-subunit of RNA polymerase	Enzymatic inactivation by ADP-ribosyltransferases (*Arr*)
			Mutations in *rpoB* encoding the β-subunit of RNA polymerase
Suppression of the respiratory chain	Bedaquiline	Inhibition of ATP synthesis	Mutation in the *atpE* (ATP synthase) gene
			Expression of the efflux pump (*Rv0678)* (*MmpS–MmpL*)
	Clofazimine	Suppression of bacterial proliferation by blocking the intracellular redox cycling	Expression of the efflux pump (*MmpS5–MmpL5*)

## Antimicrobial susceptibility tests in NTMs

Currently, in vitro antimicrobial susceptibility testing (AST) for NTMs is performed according to the recommendations of the Clinical and Laboratory Standards Institute (CLSI). The recommended standard method for AST is broth microdilution using Mueller–Hinton broth (MHB) (Griffith et al. 2007). Although broth microdilution is accepted as the standard method for growing mycobacteria on solid media, there are some studies conducted with automated systems (e.g., BACTEC) and E-test from routinely available liquid media compared with the reference method (Brown-Elliott Barbara et al. 2012).

Commercial systems based on standardized broth microdilution methods are available to speed up laboratory workflow and reduce errors in pipetting. These systems, which allow for faster and more accurate AST, are called sensititre methods. The sensititre method reduces manual operations in laboratories and provides high reproducibility and reliability (Garcia Carvalho et al. 2021). Sensititre plates are dry microdilution plates and contain lyophilized drugs, and their concentrations have been prepared and controlled by the manufacturer. After the incubation period, the plates can be read visually (manually) or automatically evaluated using equipment that allows digital capture of the image (Sangsayunh et al. 2024).

However, susceptibility tests for NTM are controversial because in most cases, there is a discrepancy between in vitro drug susceptibility and treatment response (Ryu et al. 2016). This may be due to the fact that resistance to drugs depends on a wide variety of mechanisms developed by the microorganism. In general, AST for NTM should only be performed for clinically significant isolates. The CLSI updated AST standards in 2018, and the updated guideline recommends AST for slow-growing NTMs, including MAC, *M. kansasii*, and *M. marinum*, and for fast-growing NTMs. There is insufficient data and standardized methods for other species. AST should be repeated if culture positivity persists after 3 months of antimicrobial therapy (Brown-Elliott and Woods 2019; Pennington et al. 2021).

In the broth microdilution method, cation-adjusted Mueller–Hinton broth (CAMHB) with added OADC (oleic acid, albumin, dextrose, catalase) is used for slow-growing mycobacteria, while only CAMHB is used for fast-growing mycobacteria. Twofold serial dilutions of broth and antimicrobials are prepared and added to 96-well U-bottom microplates. Finally, 1 × 10^4^–5 × 10^5^ CFU bacterial suspension is inoculated into the plates. After inoculation, the plates are incubated at 30–37 °C for 3 to 4 days for fast-growing mycobacteria and for at least 7 days for slow-growing mycobacteria (up to 14 days if no growth is detected). The last well in which no growth is detected at the end of incubation is reported as the MIC (Brown-Elliott & Woods 2019; Huang et al. 2020).

The first-line drugs for which susceptibility testing is recommended for MAC are macrolides and amikacin. In clinical studies, in vitro susceptibility testing and clinical response have been found to be related to these agents. For MAC isolates with macrolide resistance and/or isolates from patients who cannot tolerate macrolide therapy, the second-line drugs for which susceptibility testing is recommended are linezolid and moxifloxacin, despite their limited efficacy (Huang et al. 2020).

The CLSI recommends RIF and CLA susceptibility testing for *M. kansasii*. Treatment failure for *M. kansasii* is generally associated with RIF resistance. RIF-susceptible *M. kansasii* isolates do not require AST for other agents. Second-line antimicrobials that should be tested for RIF-resistant isolates include amikacin, ciprofloxacin, moxifloxacin, linezolid, rifabutin, doxycycline/minocycline, and trimethoprim-sulfamethoxazole (Huang et al. 2020).

Routine AST is not recommended for *M. marinum* because it is sensitive to the most commonly used antimicrobial agents. However, AST should be performed in patient isolates that do not respond to treatment for several months and have positive cultures. AST includes antimicrobials that should be tested in RIF-resistant *M. kansasii* isolates (Huang et al. 2020).

The CLSI-recommended antimicrobials for rapidly growing mycobacteria are amikacin, cefoxitin, ciprofloxacin, CLA, doxycycline, imipenem, linezolid, moxifloxacin, tobramycin, and trimethoprim-sulfamethoxazole. Among rapidly growing mycobacteria, MABC is the primary cause of human infections. CLSI recommends defining MABC at the subspecies level (Brown-Elliott and Woods 2019; Huang et al. 2020).

Additionally, all MABC and *M. fortuitum* isolates should be routinely tested for inducible macrolide resistance due to the presence of the *erm41* gene. CLA incubation should be extended to 14 days to detect inducible macrolide resistance. Detection of CLA resistance should be performed in *M. abscessus* pulmonary disease (Huang et al. 2020; Pennington et al. 2021).

## Conclusion

The resistance mechanisms of NTMs to antimicrobial therapies are a thick, impermeable hydrophobic cell wall that acts as the first line of defense; enzymatic modification that reduces the antimicrobial effect of the drug; target site modification that prevents drugs from reaching their target; and EPs that actively expel drug molecules from the cell. Even if the drug is effective in killing or inhibiting the growth of mycobacteria, mycobacterial colonies may persist on surfaces by forming inert biofilms or may escape the effect of the drug within granulomas within the host. Despite international treatment guidelines, treatment for NTM infections is mostly empirical and not completely successful. For clinically important NTM isolates, species-level identification and AST must be performed and treatment recommended by international guidelines should be administered.

Discovery of new effective drugs is an urgent necessity as current treatments for NTM infections, which are increasing worldwide, have proven to be long in duration, toxic, and ineffective.

## Data Availability

No new data were generated or analyzed in this study.

## References

[CR1] Alav I, Sutton JM, Rahman KM (2018) Role of bacterial efflux pumps in biofilm formation. J Antimicrob Chemother 73:2003–2020. 10.1093/jac/dky042 29506149 10.1093/jac/dky042

[CR2] AlMatar M, Albarri O, Makky EA, Köksal F (2021) Efflux pump inhibitors: new updates. Pharmacol Rep 73:1–16. 10.1007/s43440-020-00160-9 32946075 10.1007/s43440-020-00160-9

[CR3] Broncano-Lavado A, Senhaji-Kacha A, Santamaría-Corral G, Esteban J, García-Quintanilla M (2022) Alternatives to antibiotics against Mycobacterium abscessus. Antibiot (Basel) 11(10):1322 10.3390/antibiotics11101322 10.3390/antibiotics11101322 PMC9598287 36289979

[CR4] Brown-Elliott Barbara A, Nash Kevin A, Wallace Richard J (2012) Antimicrobial susceptibility testing, drug resistance mechanisms, and therapy of infections with nontuberculous mycobacteria. Clin Microbiol Rev 25:545–582. 10.1128/cmr.05030-11 22763637 10.1128/CMR.05030-11 PMC3416486

[CR5] Brown-Elliott BA, Woods GL (2019) Antimycobacterial susceptibility testing of nontuberculous mycobacteria. J Clin Microbiol 57. 10.1128/jcm.00834-19 10.1128/JCM.00834-19 PMC6760954 31315954

[CR6] De Rossi E, Aínsa JA, Riccardi G (2006) Role of mycobacterial efflux transporters in drug resistance: an unresolved question. FEMS Microbiol Rev 30:36–52. 10.1111/j.1574-6976.2005.00002.x 16438679 10.1111/j.1574-6976.2005.00002.x

[CR7] Faria S, Joao I, Jordao L (2015) General overview on nontuberculous mycobacteria, biofilms, and human infection. J Pathog 2015:809014. 10.1155/2015/809014 26618006 10.1155/2015/809014 PMC4649093

[CR8] Garcia Carvalho NF, Pedace CS, Barbosa de Almeida AR, dos Santos Simeão FC, Chimara E (2021) Evaluation of drug susceptibility in nontuberculous Mycobacteria using the SLOMYCO and RAPMYCO sensititre plates. The International Journal of Mycobacteriology 10:379–387. 10.4103/ijmy.ijmy_219_21 34916455 10.4103/ijmy.ijmy_219_21

[CR9] Gopalaswamy R, Shanmugam S, Mondal R, Subbian S (2020) Of tuberculosis and non-tuberculous mycobacterial infections - a comparative analysis of epidemiology, diagnosis and treatment. J Biomed Sci 27:74. 10.1186/s12929-020-00667-6 32552732 10.1186/s12929-020-00667-6 PMC7297667

[CR10] Griffith DE, Aksamit T, Brown-Elliott BA, Catanzaro A, Daley C, Gordin F et al (2007) An official ATS/IDSA statement: diagnosis, treatment, and prevention of nontuberculous mycobacterial diseases. Am J Respir Crit Care Med 175:367–416. 10.1164/rccm.200604-571ST 17277290 10.1164/rccm.200604-571ST

[CR11] Gutiérrez AV, Richard M, Roquet-Banères F, Viljoen A, Kremer L (2019) The TetR family transcription factor MAB_2299c regulates the expression of two distinct MmpS-MmpL efflux pumps involved in cross-resistance to clofazimine and bedaquiline in Mycobacterium abscessus. Antimicrob Agents Chemother 63:e01000-19. 10.1128/aac.01000-19 31332077 10.1128/AAC.01000-19 PMC6761555

[CR12] Helmy YA, Taha-Abdelaziz K, Hawwas HAE, Ghosh S, AlKafaas SS, Moawad MMM et al (2023) Antimicrobial resistance and recent alternatives to antibiotics for the control of bacterial pathogens with an emphasis on foodborne pathogens. Antibiotics (Basel)12. 10.3390/antibiotics12020274 10.3390/antibiotics12020274 PMC9952301 36830185

[CR13] Huang WC, Yu MC, Huang YW (2020) Identification and drug susceptibility testing for nontuberculous mycobacteria. J Formos Med Assoc 119(Suppl 1):S32-s41. 10.1016/j.jfma.2020.05.002 32423573 10.1016/j.jfma.2020.05.002

[CR14] Jacobo-Delgado YM, Rodríguez-Carlos A, Serrano CJ, Rivas-Santiago B (2023) Mycobacterium tuberculosis cell-wall and antimicrobial peptides: a mission impossible? Front Immunol 14:1194923. 10.3389/fimmu.2023.1194923 37266428 10.3389/fimmu.2023.1194923 PMC10230078

[CR15] Johansen MD, Herrmann JL, Kremer L (2020) Non-tuberculous mycobacteria and the rise of Mycobacterium abscessus. Nat Rev Microbiol 18:392–407. 10.1038/s41579-020-0331-1 32086501 10.1038/s41579-020-0331-1

[CR16] Laws M, Jin P, Rahman KM (2022) Efflux pumps in Mycobacterium tuberculosis and their inhibition to tackle antimicrobial resistance. Trends Microbiol 30:57–68. 10.1016/j.tim.2021.05.001 34052094 10.1016/j.tim.2021.05.001

[CR17] Louw GE, Warren RM, Gey van Pittius NC, McEvoy CR, Van Helden PD, Victor TC (2009) A balancing act: efflux/influx in mycobacterial drug resistance. Antimicrob Agents Chemother 53:3181–3189. 10.1128/aac.01577-08 19451293 10.1128/AAC.01577-08 PMC2715638

[CR18] Luthra S, Rominski A, Sander P (2018) The role of antibiotic-target-modifying and antibiotic-modifying enzymes in Mycobacterium abscessus drug resistance. Front Microbiol 9:2179. 10.3389/fmicb.2018.02179 30258428 10.3389/fmicb.2018.02179 PMC6143652

[CR19] Nasiri MJ, Haeili M, Ghazi M, Goudarzi H, Pormohammad A, Imani Fooladi AA, Feizabadi MM (2017) New insights in to the intrinsic and acquired drug resistance mechanisms in mycobacteria. Front Microbiol 8:681. 10.3389/fmicb.2017.00681 28487675 10.3389/fmicb.2017.00681 PMC5403904

[CR20] Nguyen TQ, Heo BE, Jeon S, Ash A, Lee H, Moon C, Jang J (2024) Exploring antibiotic resistance mechanisms in Mycobacterium abscessus for enhanced therapeutic approaches. Front Microbiol 15:1331508. 10.3389/fmicb.2024.1331508 38380095 10.3389/fmicb.2024.1331508 PMC10877060

[CR21] Pennington KM, Vu A, Challener D, Rivera CG, Shweta FNU, Zeuli JD, Temesgen Z (2021) Approach to the diagnosis and treatment of non-tuberculous mycobacterial disease. J Clin Tuberc Other Mycobact Dis 24:100244. 10.1016/j.jctube.2021.100244 34036184 10.1016/j.jctube.2021.100244 PMC8135042

[CR22] Rindi L (2020) Efflux pump inhibitors against nontuberculous mycobacteria. Int J Mol Sci 21:4191. 10.3390/ijms21124191 32545436 10.3390/ijms21124191 PMC7348771

[CR23] Rudra P, Hurst-Hess K, Lappierre P, Ghosh P (2018) High levels of intrinsic tetracycline resistance in Mycobacterium abscessus are conferred by a tetracycline-modifying monooxygenase. Antimicrob Agents Chemother 62:e00119-18. 10.1128/aac.00119-18 29632012 10.1128/AAC.00119-18 PMC5971581

[CR24] Ryu YJ, Koh WJ, Daley CL (2016) Diagnosis and treatment of nontuberculous mycobacterial lung disease: clinicians’ perspectives. Tuberc Respir Dis (Seoul) 79:74–84. 10.4046/trd.2016.79.2.74 27066084 10.4046/trd.2016.79.2.74 PMC4823187

[CR25] Sachan RSK, Mistry V, Dholaria M, Rana A, Devgon I, Ali I et al (2023) Overcoming Mycobacterium tuberculosis drug resistance: novel medications and repositioning strategies. ACS Omega 8:32244–32257. 10.1021/acsomega.3c02563 37720746 10.1021/acsomega.3c02563 PMC10500578

[CR26] Sangsayunh P, Sanchat T, Sangkaew K, Thuansuwan W (2024) Diagnostic performance of genotype MTBDRsl assay (version 2) for detecting 2nd line drug resistance tb compared to sensititre MycoTBI MIC in MDR-TB patients. J Med Assoc Thail 107. 10.35755/jmedassocthai.2024.1.13932

[CR27] Saxena S, Spaink HP, Forn-Cuní G (2021) Drug resistance in nontuberculous mycobacteria: mechanisms and models. Biology (Basel) 10. 10.3390/biology10020096 10.3390/biology10020096 PMC7911849 33573039

[CR28] Schmalstieg AM, Srivastava S, Belkaya S, Deshpande D, Meek C, Leff R et al (2012) The antibiotic resistance arrow of time: efflux pump induction is a general first step in the evolution of mycobacterial drug resistance. Antimicrob Agents Chemother 56:4806–4815. 10.1128/aac.05546-11 22751536 10.1128/AAC.05546-11 PMC3421847

[CR29] Singh P, Rameshwaram NR, Ghosh S, Mukhopadhyay S (2018) Cell envelope lipids in the pathophysiology of Mycobacterium tuberculosis. Future Microbiol 13:689–710. 10.2217/fmb-2017-0135 29771143 10.2217/fmb-2017-0135

[CR30] Singh R, Dwivedi SP, Gaharwar US, Meena R, Rajamani P, Prasad T (2020) Recent updates on drug resistance in Mycobacterium tuberculosis. J Appl Microbiol 128:1547–1567. 10.1111/jam.14478 31595643 10.1111/jam.14478

[CR31] Skinner KT, Palkar AM, Hong AL (2023) Genetics of ABCB1 in cancer. *Cancers (Basel) 15*. 10.3390/cancers15174236 10.3390/cancers15174236 PMC10487083 37686513

[CR32] Song L, Wu X (2016) Development of efflux pump inhibitors in antituberculosis therapy. Int J Antimicrob Agents 47:421–429. 10.1016/j.ijantimicag.2016.04.007 27211826 10.1016/j.ijantimicag.2016.04.007

[CR33] Tarashi S, Siadat SD, Fateh A (2022) Nontuberculous mycobacterial resistance to antibiotics and disinfectants: challenges still ahead. Biomed Res Int 2022:8168750. 10.1155/2022/8168750 35257011 10.1155/2022/8168750 PMC8898113

[CR34] Tran T, Bonham AJ, Chan ED, Honda JR (2019) A paucity of knowledge regarding nontuberculous mycobacterial lipids compared to the tubercle bacillus. Tuberculosis (Edinb) 115:96–107. 10.1016/j.tube.2019.02.008 30948183 10.1016/j.tube.2019.02.008

[CR35] van Ingen J, Boeree MJ, van Soolingen D, Mouton JW (2012) Resistance mechanisms and drug susceptibility testing of nontuberculous mycobacteria. Drug Resist Updat 15:149–161. 10.1016/j.drup.2012.04.001 22525524 10.1016/j.drup.2012.04.001

[CR36] Victoria L, Gupta A, Gómez JL, Robledo J (2021) Mycobacterium abscessus complex: a review of recent developments in an emerging pathogen. Front Cell Infect Microbiol 11:659997. 10.3389/fcimb.2021.659997 33981630 10.3389/fcimb.2021.659997 PMC8108695

[CR37] Wu ML, Aziz DB, Dartois V, Dick T (2018) NTM drug discovery: status, gaps and the way forward. Drug Discov Today 23:1502–1519. 10.1016/j.drudis.2018.04.001 29635026 10.1016/j.drudis.2018.04.001 PMC6078814

